# New insights into the photochemistry of carotenoid spheroidenone in light-harvesting complex 2 from the purple bacterium *Rhodobacter sphaeroides*

**DOI:** 10.1007/s11120-016-0322-2

**Published:** 2016-11-16

**Authors:** Dariusz M. Niedzwiedzki, Preston L. Dilbeck, Qun Tang, Elizabeth C. Martin, David F. Bocian, C. Neil Hunter, Dewey Holten

**Affiliations:** 1 Department of Chemistry, Washington University in St. Louis, St. Louis, MO 63130 USA; 2 Department of Chemistry, University of California Riverside, Riverside, CA 92521 USA; 3 Department of Molecular Biology and Biotechnology, University of Sheffield, Sheffield, S10 2TN UK; 4 Photosynthetic Antenna Research Center, Washington University in St. Louis, Campus Box 1138, St. Louis, MO 63130 USA

**Keywords:** Carotenoids, Energy transfer, Ultrafast spectroscopy, Light-harvesting complex, Purple bacteria

## Abstract

Light-harvesting complex 2 (LH2) from the semi-aerobically grown purple phototrophic bacterium *Rhodobacter sphaeroides* was studied using optical (static and time-resolved) and resonance Raman spectroscopies. This antenna complex comprises bacteriochlorophyll (BChl) *a* and the carotenoid spheroidenone, a ketolated derivative of spheroidene. The results indicate that the spheroidenone-LH2 complex contains two spectral forms of the carotenoid: (1) a minor, “blue” form with an S_2_ (1^1^B_u_^+^) spectral origin band at 522 nm, shifted from the position in organic media simply by the high polarizability of the binding site, and (2) the major, “red” form with the origin band at 562 nm that is associated with a pool of pigments that more strongly interact with protein residues, most likely via hydrogen bonding. Application of targeted modeling of excited-state decay pathways after carotenoid excitation suggests that the high (92%) carotenoid-to-BChl energy transfer efficiency in this LH2 system, relative to LH2 complexes binding carotenoids with comparable double-bond conjugation lengths, derives mainly from resonance energy transfer from spheroidenone S_2_ (1^1^B_u_^+^) state to BChl *a* via the Q_x_ state of the latter, accounting for 60% of the total transfer. The elevated S_2_ (1^1^B_u_^+^) → Q_x_ transfer efficiency is apparently associated with substantially decreased energy gap (increased spectral overlap) between the virtual S_2_ (1^1^B_u_^+^) → S_0_ (1^1^A_g_^−^) carotenoid emission and Q_x_ absorption of BChl *a*. This reduced energetic gap is the ultimate consequence of strong carotenoid–protein interactions, including the inferred hydrogen bonding.

## Introduction

Carotenoids are a class of chemical compounds that are naturally synthesized by photosynthetic organisms. One primary function is to provide absorption in the green spectral region, thereby complementing the light-harvesting capacity of the (bacterio)chlorin pigments. The absorption spectrum of carotenoids typically appears in the spectral range between 400 and 550 nm and is associated with the strongly allowed S_0_ (1^1^A_g_^−^) → S_2_ (1^1^B_u_^+^) electronic transition. The lowest excited state, S_1_ (2^1^A_g_^−^), is optically silent because the one-photon transition from S_0_ does not satisfy the requisite change in state symmetry (g ↔ u) and pseudoparity (+ ↔ −) (Christensen 1999). The energies of both the S_1_ and S_2_ excited states depend on carbon–carbon double-bond (C=C) conjugation length (*N*_C=C_); therefore, the more extended the π-electron system of the carotenoid, the lower the energies of its excited states.

Photosynthetic purple bacteria produce a subclass of the molecules commonly referred to as open-chain carotenoids and incorporate them into key photosynthetic machinery such as light-harvesting (LH) complexes and reaction centers. A major fraction of the carotenoid pool is incorporated into cyclic antenna complexes LH1 and LH2 along with bacteriochlorophyll (BChl) *a* molecules (Gabrielsen et al. 2009). The LH2 are peripheral complexes that transfer energy to the LH1 complexes, which in turn transfer energy to the reaction centers (RC) embedded therein. Numerous investigations have probed the role(s) of carotenoids in the peripheral LH2 antenna complexes from various bacterial strains that contain a number of different carotenoids. Naturally produced LH2 complexes tend not to diversify the carotenoid portfolio but mainly incorporate representatives with low and medium conjugation *N*_C=C_ between 9 and 11. Prior studies have demonstrated that for those carotenoids the carotenoid-to-BChl *a* energy transfer efficiency (*Φ*_Car→BChl_) is considerable (>60%), thereby supporting light harvesting by absorbing light in a spectral range not accessible to BChl *a*. The highest *Φ*_Car→BChl_ yields of ~95% are observed in LH2s accommodating carotenoids with 9–10 C=C bonds such as neurosporene (*N*_C=C_ = 9) or spheroidene (*N*_C=C_ = 10) (Angerhofer et al. 1995; Chi et al. 2014; Cong et al. 2008; Frank and Cogdell 1996). On the other hand, elongation of conjugation to *N*_C=C_ = 11 (e.g., rhodopin or lycopene) causes a substantial drop in the *Φ*_Car→BChl_ efficiency to ~50–60% (Angerhofer et al. 1995; Billsten et al. 2002; Cong et al. 2008; Frank and Cogdell 1996; Garcia-Asua et al. 2002).

Interestingly, such a drop in the *Φ*_Car→BChl_ efficiency is not observed if the eleventh double bond is a C=O bond as in spheroidenone. This carotenoid is a simple derivative of spheroidene and is naturally produced and incorporated into the LH2 and LH1 complexes in wild-type *Rhodobacter* (*Rba.*) *sphaeroides* grown either semi-aerobically in the dark, or photosynthetically in the presence of low levels of oxygen. Previous studies have shown that *Φ*_Car→BChl_ is nearly 100% in spheroidenone-LH2 complexes and that spheroidenone (*N* = *N*_C=C_ + *N*_C=O_ = 10 + 1 = 11) performs equally well or even better than neurosporene (*N*_C=C_ = 9) or spheroidene (*N*_C=C_ = 10) (Cong et al. 2008). Substantial prior work has suggested that the efficiency of energy transfer is determined in large part by the energies of the carotenoid S_2_ (1^1^B_u_^+^) and S_1_ (2^1^A_g_^−^) excited states with respect to the energies of the BChl *a* S_2_ (Q_x_) and S_1_ (Q_y_) excited states (Cong et al. 2008; Dilbeck et al. 2016; Niedzwiedzki et al. 2015; Papagiannakis et al. 2003; 2006; Polivka et al. 2002; Polli et al. 2006; Rondonuwu et al. 2004).

It is well known that the energetic shift resulting from addition of a C=O bond to a carotenoid framework is not equal to that induced by C=C bond. Moreover, the energies of the S_1_ (2^1^A_g_^−^) and S_2_ (1^1^B_u_^+^) excited states are affected differently (Niedzwiedzki et al. 2015). Addition of a C=O bond has a smaller effect on the S_1_ (2^1^A_g_^−^) energy than a C=C bond, but the reverse is true for the S_2_ (1^1^B_u_^+^) state. Consequently, it is not straightforward to predict how spheroidenone should perform in the LH2 protein in comparison with carotenoids with the same nominal conjugation *N* but comprising only C=C bonds. The S_1_ (2^1^A_g_^−^) state of spheroidenone lies in the 12,800–13,000 cm^−1^ range (Cong et al. 2008; Zigmantas et al. 2004), only marginally higher than the S_1_ (2^1^A_g_^−^) state energy of open-chain carotenoids with *N* = *N*_C=C_ = 11 such as lycopene or rhodopin (12,400–12,800 cm^−1^) (Billsten et al. 2002; Niedzwiedzki et al. 2011b). If energetic considerations are of paramount importance for energy transfer, a substantial difference in *Φ*_Car→BChl_ among these carotenoids is not expected.

Recent studies of the RC-LH1-PufX complex from *Rba. sphaeroides* and *Roseobacter* (Šlouf et al. 2012, 2013) containing spheroidenone revealed other interesting properties of this carotenoid. It was proposed that the ability of the bacterium to convert spheroidene to spheroidenone when stressed by a higher level of oxygen in the environment may be a photoprotective adaption because spheroidenone seems better able than spheroidene to serve as a singlet-oxygen scavenger. In addition, it was proposed that while the spheroidenone is bound in LH1, the carotenoid’s structure is altered such that the –C=O group is in s-*trans* orientation with respect to the rest of the double-bond conjugated system. This conformational change, most likely stabilized by a hydrogen bond to a protein residue, enhances formation of the intramolecular charge transfer (ICT) excited state of the carotenoid (Šlouf et al. 2012). Such a state, or state with mixed ICT and S_1_ (2^1^A_g_^−^) character, is thought to be very efficient in energy transfer to BChl *a* in LH complexes containing keto-carotenoids such as peridinin or fucoxanthin (Bautista et al. 1999; Frank et al. 2000; Kosumi et al. 2009; 2011; Shima et al. 2003; Zigmantas et al. 2004).

In contrast to binding into RC-LH1-PufX, it has been suggested that incorporation of spheroidenone into LH2 does not alter the structure of the carotenoid and that the ICT is not formed (Šlouf et al. 2012; 2013). However, partial ICT character of the lowest singlet excited singlet state of spheroidenone in LH2 (e.g., a state with mixed ICT and S_1_ (2^1^A_g_^−^) character) would be in line with the superior performance of spheroidenone-to-BChl *a* energy transfer noted above. As this issue has not been investigated in detail, the focus of the studies described herein was to (1) examine whether the photochemical properties of spheroidenone are affected upon binding into the LH2 protein and (2) gain further insights into how spheroidenone augments the light-harvesting function of BChl *a*. To these ends, a number of static and time-resolved spectroscopic techniques at room temperature (RT) and 77 K have been employed to investigate the properties of spheroidenone in an organic medium and in the *Rba. sphaeroides* LH2 complex.

## Materials and methods

### Isolation and purification of LH2

The *Rba. sphaeroides* 2.4.1 strain was grown semi-aerobically as described by Chi et al. (Chi et al. 2014). After reaching stationary stage, the cultures were harvested and pelleted by centrifugation and then resuspended in buffer A (20 mM tris(hydroxymethyl)-aminomethane (Tris) at pH = 8.0). The photosynthetic membranes were released by ultrasonication and subsequently pelleted by centrifugation. The pelleted material was resuspended in buffer A to optical density OD_850_ ≈ 20 (1 cm path), mixed with lauryl dimethylamine-oxide (LDAO) to a final concentration of ~0.5% (v/v) and gently stirred for 20 min at room temperature (RT). The insoluble material was removed by centrifugation. The complexes were purified by loading the supernatant on an anion exchange column (Q Sepharose High Performance, GE Healthcare) equilibrated with buffer A containing 0.06% LDAO followed by 50 mM gradient steps of NaCl from 150 and 500 mM. The protein-containing fraction was eluted with 300–400 mM NaCl.

### Steady-state absorption and fluorescence spectroscopy

All steady-state absorption measurements were performed using a Shimadzu UV-1800 spectrophotometer. The absorbance of the sample was set at ~0.5, at the maximum of the carotenoid absorption band, in a 2 mm path cuvette. For low-temperature measurements, the buffer A (0.06% LDAO)-LH2 solution was mixed with glycerol in a 1:1 (v/v) ratio, transferred to a 1-cm square plastic cuvette and frozen in a VNF-100 liquid nitrogen cryostat (Janis, Woburn, MA, USA). Cryogenic studies on spheroidenone were performed in 2-methyltetrahydrofuran (2-MTHF) glass at 77 K. Fluorescence excitation spectra were recorded at RT using a Horiba-Spex NanoLog fluorometer. The excitation and detection bandwidths were set to 2–4 nm, and fluorescence was measured at a right angle from the light source incident on the sample with absorbance ≤0.1 at the emission wavelength (~890 nm).

### Resonance Raman (RR) spectroscopy

The RR spectra of spheroidene and spheroidenone in tetrahydrofuran (THF) and in the LH2 complexes were acquired at 4 °C as described in detail previously (Niedzwiedzki et al. 2015).

### Time-resolved absorption and fluorescence spectroscopy

Time-resolved pump-probe absorption experiments were carried out using a Helios femtosecond transient absorption (TA) spectrometer (Ultrafast Systems LCC, Sarasota, FL) coupled to a Spectra-Physics femtosecond laser system described previously (Niedzwiedzki et al. 2012). In order to enhance transparency in the near-infrared (NIR) region for LH2 sample, the water-based buffer was replaced with the D_2_O-based analog. The pump wavelength was set to preferentially excite the carotenoid at 520 nm or at 565 nm, the two wavelengths used for comparative purposes. The energy of the excitation beam was kept between 200 and 400 nJ at RT, corresponding to an intensity of ~0.5 – 1 × 10^14^ photons cm^−2^. For 77 K studies, in order to minimize permanent photobleaching of the sample, the energy of the pump was lowered to 100 nJ (~3 × 10^13^ photons cm^−2^).

Time-correlated single-photon counting (TCSPC) measurements were performed using a Simple-Tau 130 system (Becker–Hickl, Germany) equipped with a PMC-100-20 detector, coupled to the ultrafast laser system (Spectra-Physics, USA), as previously described (Dilbeck et al. 2016). The LH2 complexes were re-suspended to an absorbance of ≤0.1 at the B850 maximum, and the emission signal was recorded at right angle in respect to the excitation beam.

### Data processing and fitting

Group velocity dispersion in the TA datasets was accounted for using Surface Xplorer Pro software provided by Ultrafast Systems by building a dispersion correction curve from a set of initial times of transient signals obtained from single wavelength fits of representative kinetics. Directed kinetic modeling (target analysis) of transient absorption results was performed using CarpetView (Light Conversion Ltd., Vilnius, Lithuania). If the fitting model reflects the true/anticipated decay pathways following excitation of the carotenoid, it should reconstruct spectro-temporal components associated with particular processes. In principle, the TA signal (ΔA) at any delay time can be decomposed to superposition of *n*th *C*_*i*_(*t*)·Δ*A*_*i*_(*λ*) products (van Stokkum et al. 2004):

1 $$\Delta A(t,\lambda ) = \sum\limits_{i = 1}^{n} {C_{i} (t)\Delta A_{i} (\lambda )}$$

The Δ*A*_*i*_(*λ*) component is defined as the species associated difference spectrum (SADS) with temporal characteristics (rise and decay) defined by concentration *C*_*i*_(*t*). The concentration of the initial Δ*A*_*i*_(*λ*) is convoluted by the instrument response function (IRF). Spectroscopically, each SADS does not simply reflect TA of a specific molecular species but also carries associated bleaching of a ground-state absorption of contributing molecule: Δ*A*_*i*_(*λ*) = *A*_*i*_(*λ*) − *A*_*0*_(*λ*) in which *A*_*0*_(*λ*) is a ground-state absorption and *A*_*i*_(*λ*) is a transient signal associated with pure spectroscopic species. For example for spheroidenone, SADS associated with the S_1_ (1^1^A_g_^−^) state will be composite of the (S_1_ (1^1^A_g_^−^) → S_*n*_) excited-state absorption (ESA) and the ground-state absorption bleaching (GSB). The IRF was assumed to have Gaussian-like shape with the full width at half maximum (FWHM) of ~200 fs and was used as a fixed fitting parameter.

## Results

### Steady-state absorption and fluorescence of LH2 complexes

**Fig. 1:**
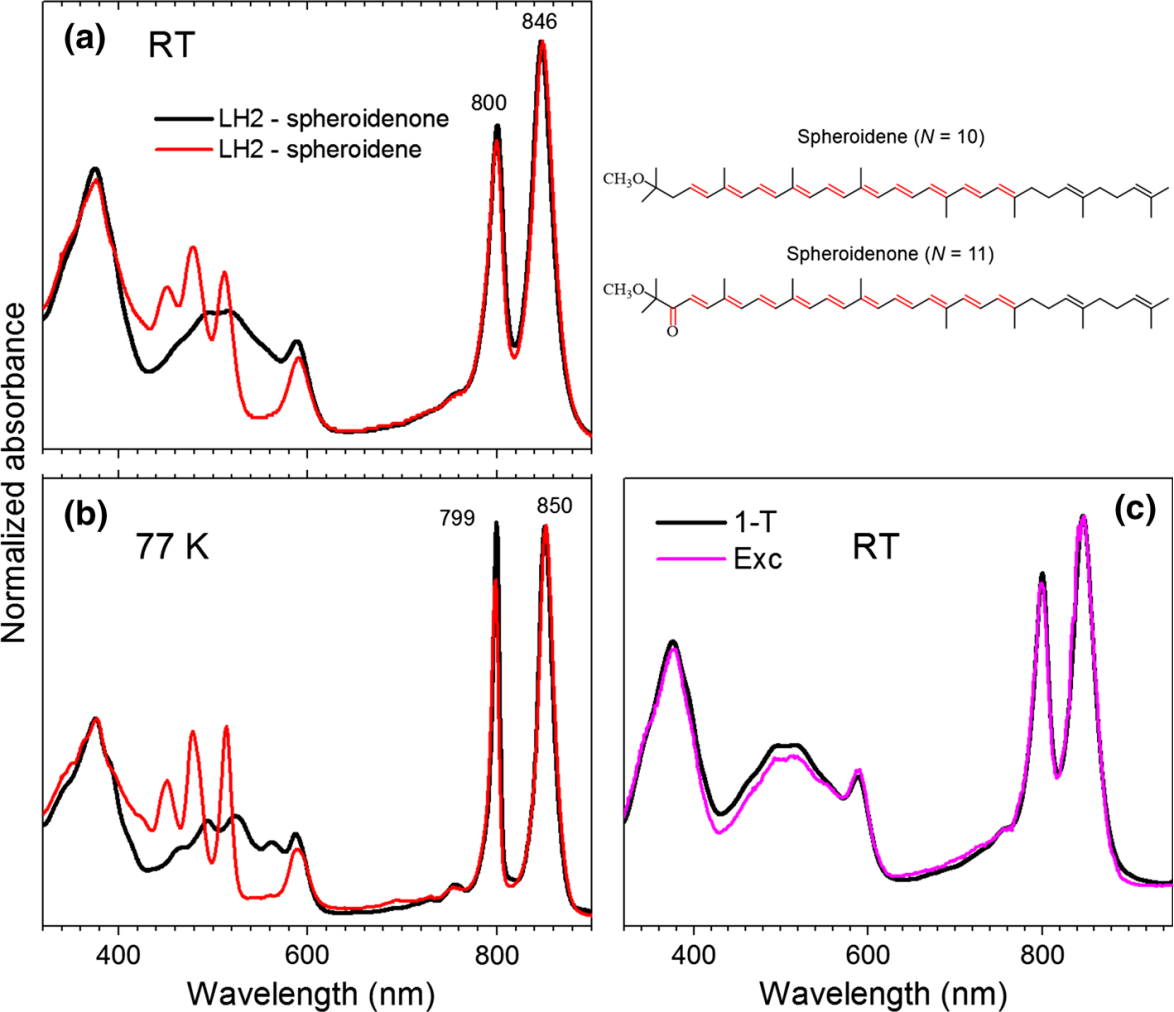
Steady-state absorption and fluorescence spectroscopy results of the spheroidene- and spheroidenone-LH2 from wild-type *Rba. sphaeroides* along with molecular structures of spheroidene and spheroidenone with highlighted nominal double-bond conjugation *N*. **a** Room temperature and **b** 77 K absorption spectra of the spheroidene- and spheroidenone-LH2. The spectra are normalized at the B850 band. **c** Absorptance (1 − *T*, where *T* is transmittance) and fluorescence excitation (Exc) profiles of the spheroidenone-LH2 sample taken at RT. The profiles are normalized at the B850 band upon assumption of 100% energy transfer efficiency. The carotenoid exhibits an overall 92% efficiency of energy transfer to the BChls and produces excited B850 (B850*), which gives rise to the measured fluorescence

The RT and 77 K absorption spectra of the LH2 complexes containing spheroidene and spheroidenone, absorptance (1 − *T*, where *T* is transmittance) and fluorescence excitation (Exc) profiles of the spheroidenone-LH2 at RT are shown in Fig. 1. For comparative purposes the absorption spectra are normalized at the B850 maxima. At RT the B800 and B850 bands appear at 800 and 850 nm, respectively, in both complexes and those positions are only marginally affected by cryogenic temperature. The carotenoid absorption bands are well resolved for the spheroidene-LH2 complex, with three vibronic peaks at 511, 478, and 451 nm at RT. The positions are essentially the same at 77 K (513, 478 and 451 nm) although the bands are narrower.

The absorption contour for spheroidenone-containing LH2 is quite diffuse at RT, leading to ambiguity in the precise positions and shapes of the underlying vibronic features. Lowering the temperature to 77 K does not substantially improve the vibronic resolution, although the rough positions of the vibronic peaks can be recognized, with the (0–0) band at 562 nm. The origin feature at this position was previously observed in the cryogenic absorption and fluorescence excitation spectra of spheroidenone-containing LH2 from the same wild-type strain of *Rba. sphaeroides* (Chi et al. 2014; Cong et al. 2008; Fowler et al. 1997). This finding is significant because in order to be consistent with the position of the S_0_ → S_2_ (0–0) band for LH2-bound carotenoids having comparable conjugation length (*N*_C=C_ = 11), such a substantial spectral shift of the absorption band from the position in organic media (induced only by a highly polarizable protein environment) is not expected (vide infra).

**Fig. 2:**
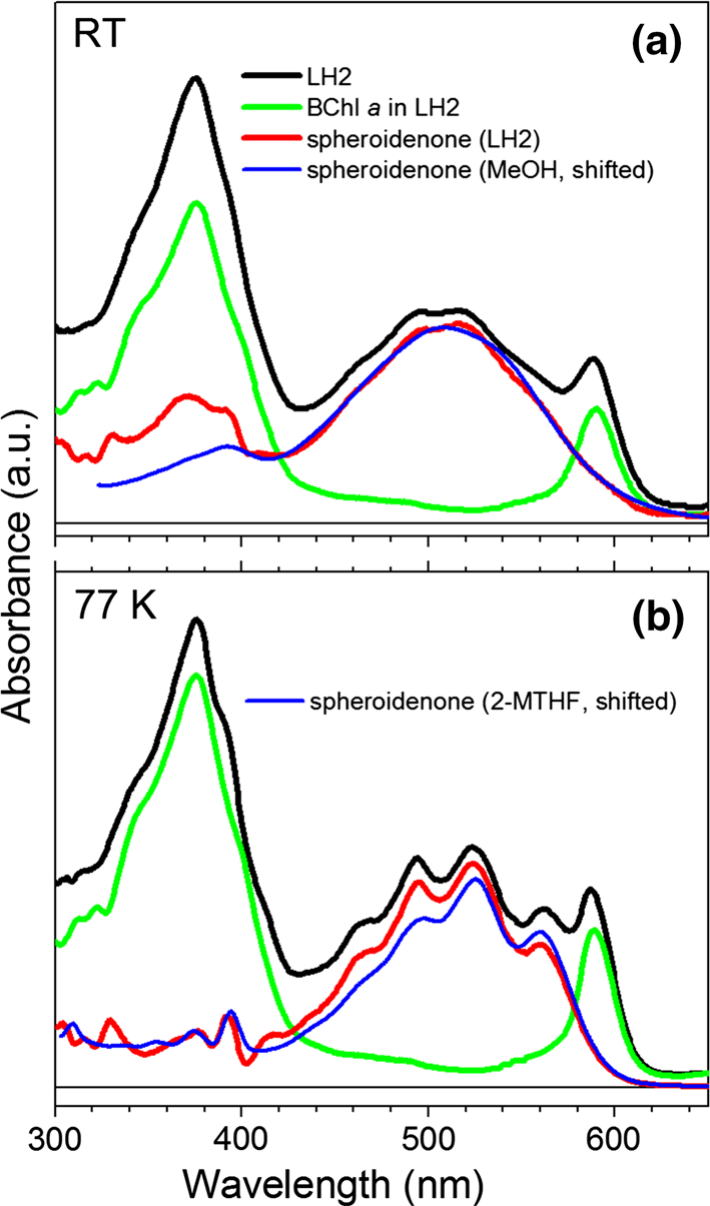
Spectral reconstruction of spheroidenone absorption in the LH2 complex at **a** RT and **b** at 77 K. The *green line* profile is an anticipated absorption spectrum of a carotenoidless LH2. The *blue* profiles are spheroidenone absorption spectra in methanol (MeOH) and in 2-MTHF shifted to lower energies for best coincidence

The absorption spectrum of the LH2-bound spheroidenone (Fig. 2) could be reconstructed by subtraction of the anticipated absorption of the LH2-bound BChl *a*, as performed in previous work (green line in Fig. 2a) (Niedzwiedzki et al. 2015). For non-keto-carotenoids, the resulting spectra, if adequately shifted in energy, track agreeably the absorption spectra taken in organic solvents, regardless of variation in solvent polarity or polarizability (Dilbeck et al. 2016). However, the situation is apparently not so straightforward for keto-containing carotenoids such as spheroidenone. The absorption spectrum of spheroidenone in nonpolar solvents with low polarizability such as *n*-hexane shows the (0–0) vibronic band at 513 nm. Changing the environment to a polar solvent such as acetonitrile or methanol induces significant spectral changes including diminished resolution of the vibronic bands and asymmetric broadening toward longer wavelengths (Frank et al. 2000). Because the absorption spectrum of spheroidenone in LH2 shows substantially diminished of vibronic resolution (Fig. 1a), it was mimicked (Fig. 2a) by the spectrum in methanol (with application of a shift in energy/wavelength). However, the solvent and LH2-bound spheroidenone spectra do not completely agree, and the presence of vibronic bands in the spectrum of the protein-bound carotenoid suggests that the polarity of the binding pocket is somewhere between the two extremes. Due to the slightly improved vibronic resolution at 77 K (Fig. 1b), the absorption spectrum of spheroidenone in LH2 actually can be better positioned and reconstructed using the absorption spectrum taken in 2-MTHF (a medium polarity solvent) at 77 K, again with a shift in energy/wavelength to obtain the best registration (Fig. 2b). However, differences in the positions and relative intensities of the vibronic features for the carotenoid in solvent (shifted) and in LH2 are evident.

**Fig. 3:**
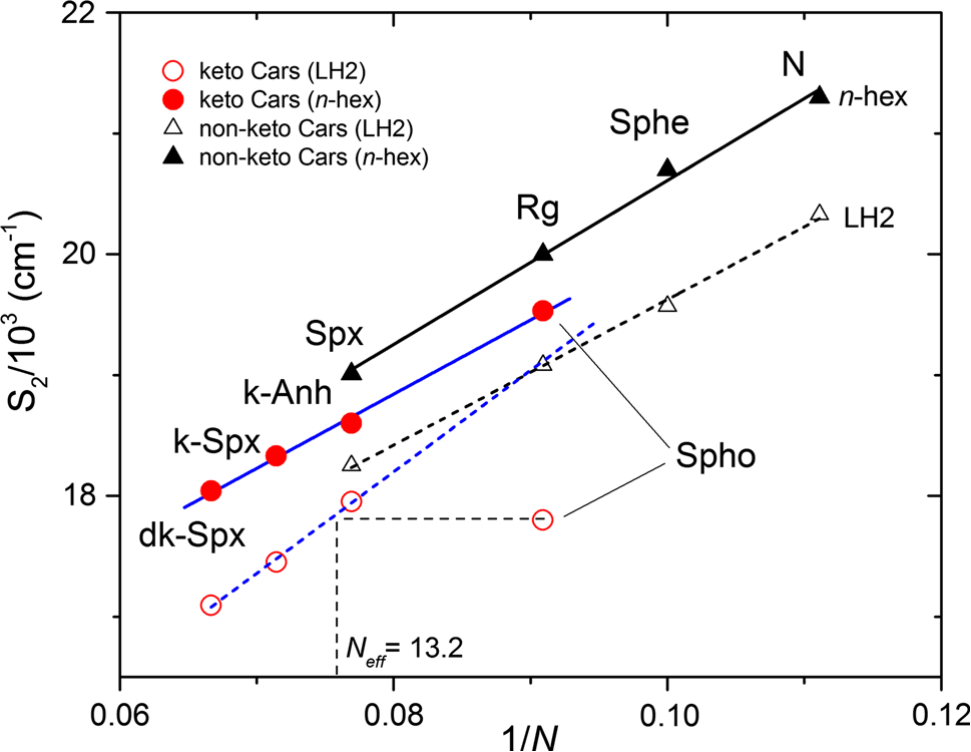
S_2_ (1^1^B_u_^+^) state energy of open-chain carotenoids found in various LH2 complexes as function of their conjugation *N* = *N*_*C*=*C*_ + *N*_*C*=*O*_. The data are provided for two environments: *n*-hexane and LH2 protein for non-keto- and keto-carotenoids. The LH2-bound spheroidenone vibronic band at 562 nm (~17,800 cm^−1^) substantially deviates from the expected pattern. *N* neurosporene, *Sphe* spheroidene, *Rg* rhodopin glucoside, *Spx* spirilloxanthin, *k*-*Anh* keto-anhydrorhodovibrin, *k*-*Spx* keto-spirilloxanthin, *dk*-*Spx* diketo-spirilloxanthin, *Spho* spheroidenone

The above considerations provide an additional framework for analyzing the unexpected finding that the (0–0) vibronic band of spheroidenone in LH2 is at 562 nm. The average energetic shift of the absorption spectrum of open-chain carotenoids (with or without keto groups) bound in the highly polarizable LH2 protein environment versus in low polarizability solvents such as *n*-hexane ranges from 800 to 1050 cm^−1^ (Cong et al. 2008; Niedzwiedzki et al. 2015). However, the spectral shift for spheroidenone is substantially larger. If the absorption of spheroidenone in *n*-hexane is used as the reference, the shift necessary to match the (0–0) vibronic band position is ~1750 cm^−1^. If a broader absorption spectrum taken in a polar solvent such as methanol is used, the shift necessary to obtain the best coincidence between spectra is ~1300 cm^−1^, still considerably larger than expected. This anomaly is visualized in Fig. 3, which plots the S_2_ (1^1^B_u_^+^) state energy (as the (0,0) absorption band position) of open-chain carotenoids present in various LH2 complexes as a function of double-bond conjugation length. The state energies of keto-carotenoids (except spheroidenone) were taken from our previous study (Dilbeck et al. 2016). Data are provided for two environments (*n*-hexane and LH2) and the two types of open-chain carotenoids (with and without keto groups). Evidently the two subgroups follow different relationships of state energy versus conjugation length and different associated trendlines. In *n*-hexane, the energy of the S_2_ (1^1^B_u_^+^) state of spheroidenone appears in the expected range and falls at the extrapolated position of the linear fit with the other three representatives with longer conjugation lengths (Niedzwiedzki et al. 2015). Extrapolation of the fit for the keto-containing carotenoids in LH2 suggests that S_2_ (1^1^B_u_^+^) state should have a (0,0) band position similar to the non-keto-carotenoid rhodopin glucoside at ~522 nm (Cong et al. 2008; Polivka et al. 2002). However, the experimental position of 562 nm is far from the anticipated value and is more characteristic of a keto-carotenoid with an effective conjugation between 13 and 14 (Fig. 3). These considerations strongly suggest that other factors in addition to a high polarizability of the protein binding pocket are responsible for the anomalous, unusually low position of the S_2_ (1^1^B_u_^+^) (0, 0) band of this *N* = 11 carotenoid.

### Resonance Raman (RR) spectroscopy

**Fig. 4:**
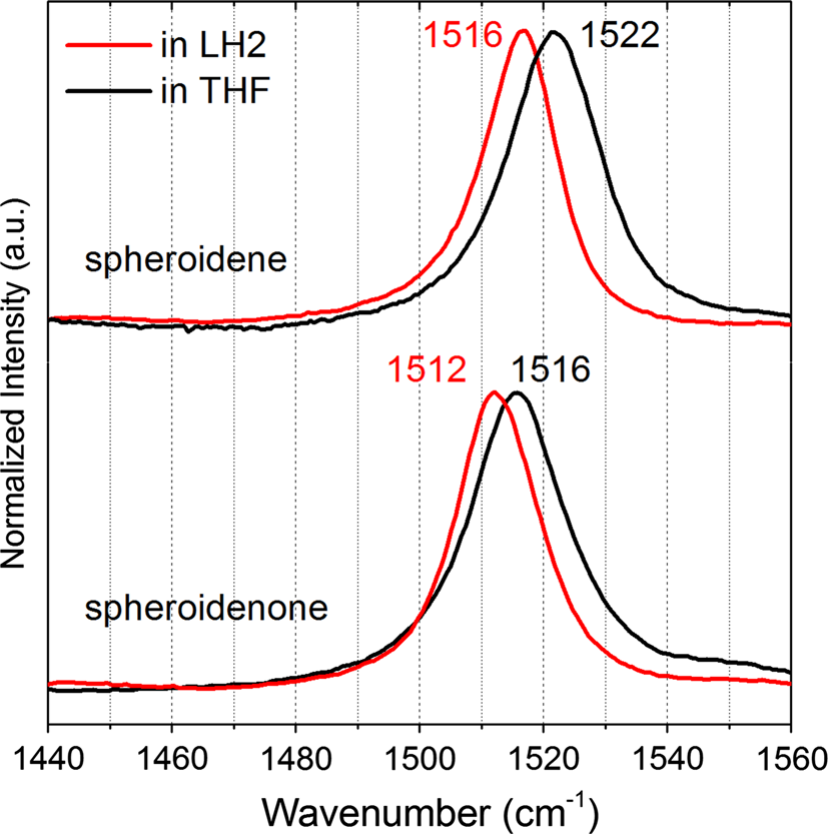
Resonance Raman spectroscopy of spheroidene and spheroidenone dissolved in THF and bound into the LH2 obtained upon excitation at 532 nm at 4 °C. The RR spectra are narrowed to *ν*_1_ mode range characteristic of C = C stretching modes of the central part of carotenoid conjugation

To further investigate how terminal attachment of a keto group to the C=C bond framework affects carotenoid conjugation, we performed a comparative RR spectroscopy of spheroidene and spheroidenone in THF solvent and in the LH2 antenna (Fig. 4). Before describing the results, some points of reference from prior studies are useful. Similar studies on β-carotene (no keto group) and canthaxanthin (two keto groups) in benzene showed that terminal keto groups have little (1 cm^−1^) effect on the *ν*_1_ mode energy (Okamoto et al. 1997). However, because the keto groups in canthaxanthin are located in rather rigid β-rings their influence on the main backbone conjugation might be different than for spheroidenone. In particular, for open-chain carotenoids such as diketospirilloxanthin, the two terminally attached keto groups result in a significant downshift of the *ν*_1_ mode energy of 6 cm^−1^ with respect to spirilloxanthin, the parent carotenoid with the same number of C=C double bonds (Dilbeck et al. 2016).

The RR results of spheroidene and spheroidenone given in Fig. 4 show energies characteristic of the *ν*_1_ band that arise from C=C stretching motions, mostly involving the central part of the carotenoid skeleton (Merlin 1985). Terminal attachment of a keto group to the C=C framework results in a substantial downshift (6 cm^−1^ in THF) shift of the *ν*_1_ band in spheroidenone versus spheroidene, the same as the above-noted shift found previously for the spirilloxanthin–diketospirilloxanthin pair (Dilbeck et al. 2016). Evidently attachment of keto-groups results in some redistribution of bond lengths along the C=C conjugation pathway that affect the modes/energies. From the standpoint of previous RR studies on open-chain carotenoids, spheroidenone behaves like lycopene with 11 C=C double bonds, (Dilbeck et al. 2016) indicating that reliance on RR shifts to compare conjugation lengths in keto- versus non-keto-carotenoids is not necessarily straightforward. Upon binding spheroidenone into LH2, the *ν*_1_ band shift for spheroidenone versus spheroidene is slightly reduced to 4 cm^−1^. Band shifts of this magnitude are typical for other carotenoids upon binding to LH2, suggesting that the primary effect is an increase in the polarizability of the carotenoid environment (Mendes-Pinto et al. 2013).

### Efficiency of energy transfer from spheroidenone to the BChls

The overall efficiency of energy flow from excited carotenoid to the BChls (B800 and B850) to produce the lowest singlet excited state of the BChl *a* array B850* was assessed from the steady-state data shown in Fig. 1. The fluorescence from B850* was monitored at 920 nm, and the excitation spectrum of the fluorescence obtained (Fig. 1c, magenta). The fluorescence excitation spectrum was compared with the absorptance (1 − *T*, where *T* is the transmittance) spectrum (Fig. 1c, black). The two spectra were normalized at the B850 band, corresponding to a 100% yield of fluorescence for each photon absorbed (i.e., a unity “energy transfer” efficiency). The ratio of amplitudes of the fluorescence excitation features to the 1 − *T* features across the carotenoid absorption profile (450–550 nm) afforded *Φ*_Car→BChl_ = 0.92, or a percent efficiency of 92%. This value is in good agreement with prior measurements (Chi et al. 2014; Cong et al. 2008).

### Transient absorption studies of spheroidenone in organic media

**Fig. 5:**
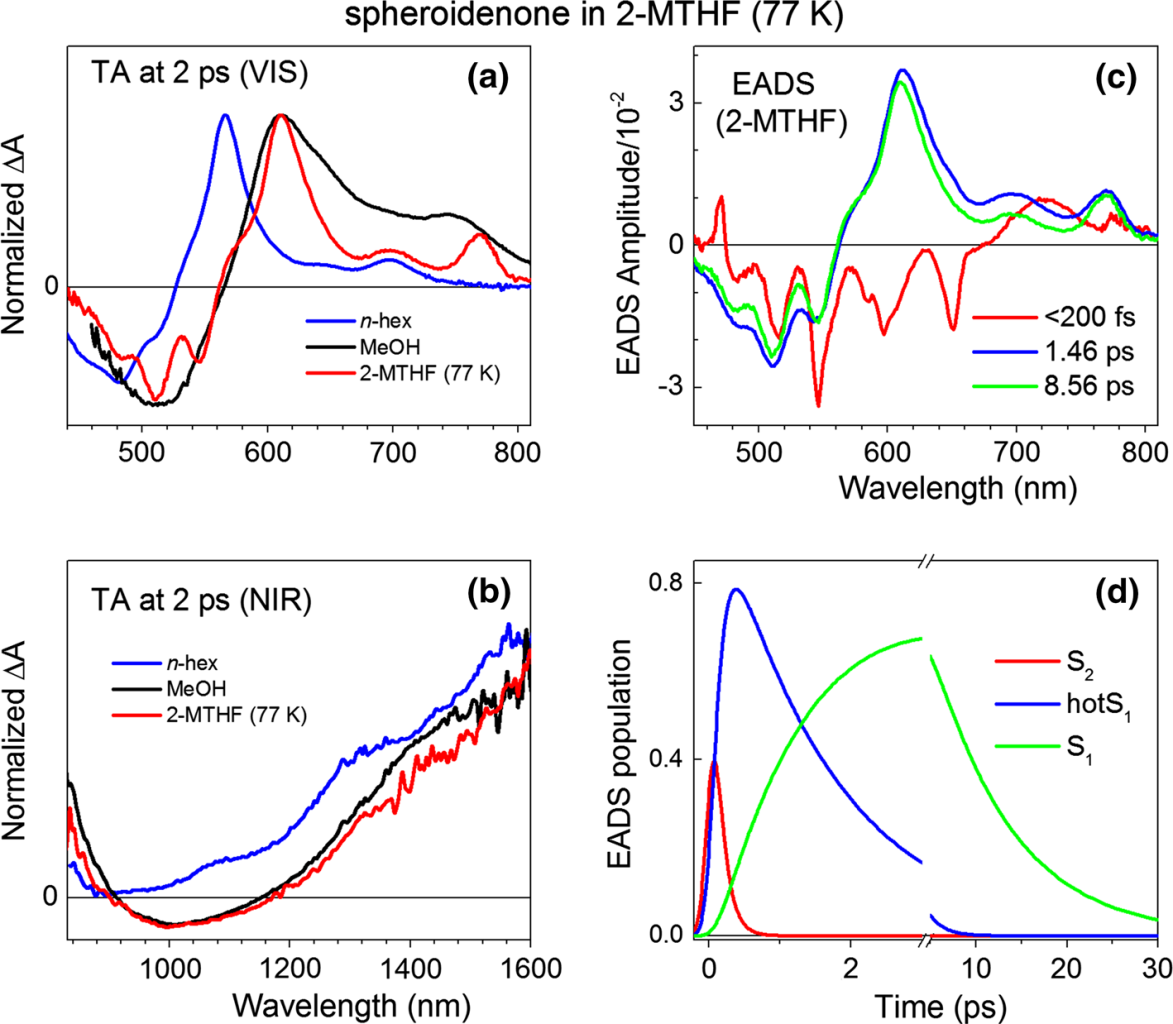
Comparison of transient absorption of spheroidenone in 2-MTHF at 77 K with polar and nonpolar solvents at RT. **a** Representative TA spectra taken in the VIS range 2 ps after excitation, **b** representative TA spectra taken in the NIR range 2 ps after excitation. Spectra were normalized to their maxima for easier evaluation, **c** Evolution associated difference spectra (EADS) amplitudes and **d** concentrations obtained from applying global analysis according to sequential decay

There have been several TA studies of spheroidenone in solvents with various polarities and polarizabilities at RT (Cong et al. 2008; Frank et al. 2000; Niedzwiedzki et al. 2011a; Zigmantas et al. 2004). Thus we focus here on results obtained at cryogenic temperature, conditions under which this carotenoid has not been studied in detail previously. Representative TA spectra in the visible (VIS) and NIR spectral ranges in three exemplary solvents with similar polarizabilities but vastly different polarities: *n*-hexane (nonpolar), methanol (very polar) (both RT) and glass forming 2-MTHF (mid-polar) (77 K) are given in Fig. 5a, b. The effect of the reduction in temperature (as well larger polarizability of the glass) on excited state properties is clearly seen in the VIS spectral range. The S_1_ (2^1^A_g_^−^) → S_*n*_ excited state absorption (ESA) bands are bathochromically shifted at 77 K versus RT. The band shift is associated with narrowing of the energetic gaps between the initial (S_1_) and terminal (S_*n*_) states, driven by reduction in energy of the B_u_-like S_*n*_ states in the high polarizability solvent glass, while the energy of the S_1_ (2^1^A_g_^−^) state is relative unchanged (from unfrozen solvent).

Unlike the findings in the VIS spectra, the TA spectra in the NIR for spheroidenone in methanol at RT are very similar to those in 2-MTHF at 77 K (Fig. 5b). Under both conditions, the TA profiles in the NIR are rather broad and featureless, and reveal a negative feature between 900 and 1200 nm. Such a feature is typically observed for carotenoids for which there is a change in the character/lifetime of the lowest excited state with solvent polarity. In particular, the negative feature is typically associated with stimulated emission from an ICT state, or a state with mixed ICT and S_1_ (2^1^A_g_^−^) character, that is produced during relaxation from the upper excited states (Zigmantas et al. 2001, 2003, 2004). The presence of such a feature for spheroidenone suggests that its lowest excited state possesses some ICT character in moderately polar (2-MTHF) and strongly polar (methanol) solvents.

The results of global fitting performed (using a sequential decay routine) on the dataset for spheroidenone in 2-MTHF at 77 K are given in Fig. 5c. A satisfactory fit of the data was achieved with three spectral-kinetic components (evolution-associated difference spectra, EADS) having lifetimes of <200 fs, ~1.5 and ~8.6 ps which show that cryogenic temperature does not significantly change the excited-state dynamics. The first kinetic component (<200 fs EADS) is associated with decay of the S_2_ (1^1^B_u_^+^) state and comprises ground-state absorption bleaching (GSB) mixed with strong solvent-derived signals (stimulated Raman emission bands, etc.) that cannot be separated due to limited time resolution. Subtle changes observed in the spectral shapes of the second and third EADS suggest that ~1.5 ps EADS is associated with relaxations within the S_1_ (2^1^A_g_^−^) state. As time evolves (8.6 vs. 1.5 ps EADS), there is a small hypsochromic shift of the excited-state absorption (ESA) band (~610 nm) and some sharpening in the GSB. As EADS amplitudes do not necessarily reflect the true contribution of that spectral-kinetic component to the raw TA spectra, EADS concentrations are also provided in Fig. 4d. In order to reconstruct relative contribution of a particular EADS in TA spectrum every EADS should be multiplied by the value of its concentration at a given time and then compared.

### Transient absorption of spheroidenone in LH2 complex

**Fig. 6:**
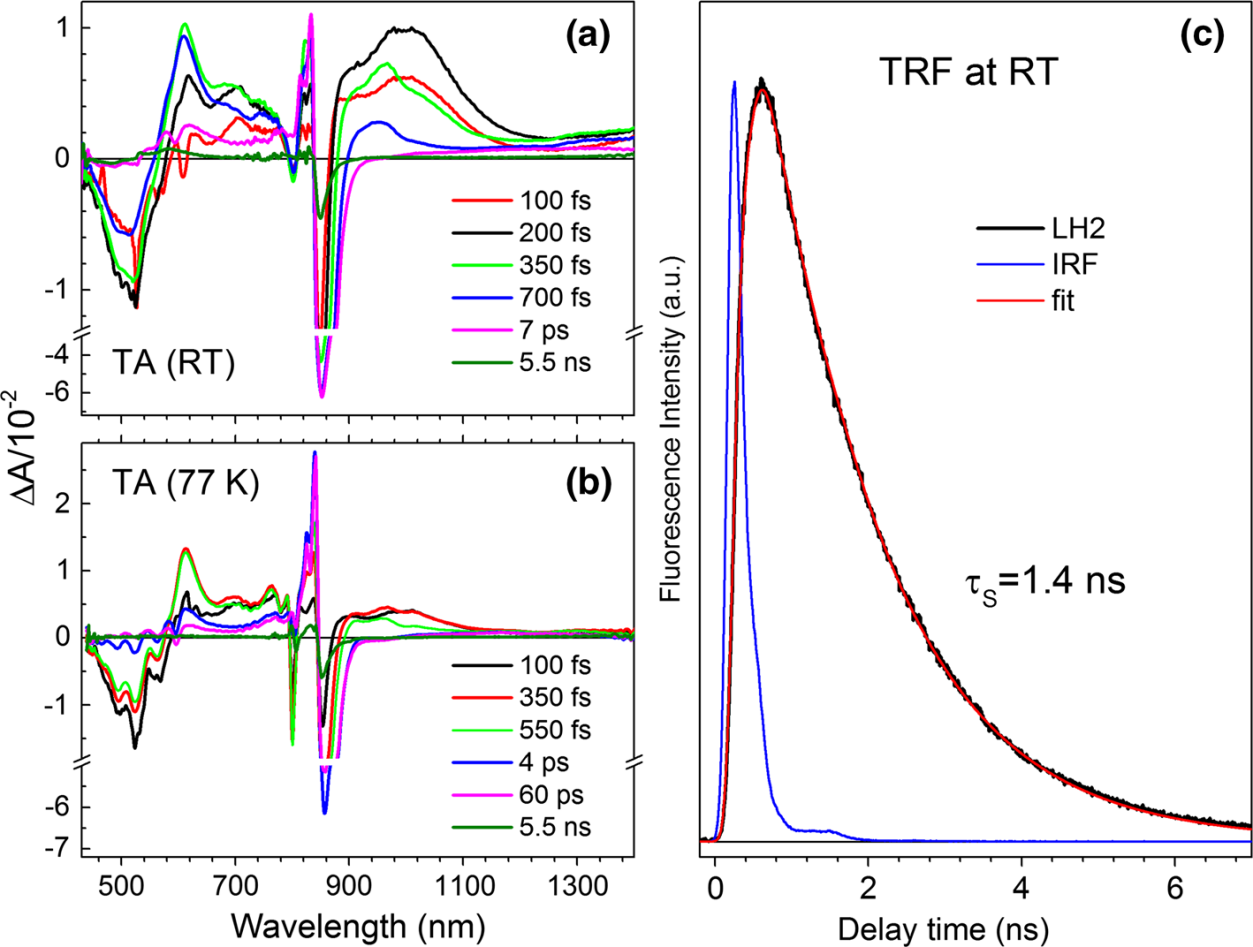
Time-resolved spectroscopy of the spheroidenone-LH2. **a** RT and **b** 77 K transient absorption spectra recorded in the visible–NIR spectral range upon excitation into the carotenoid band at 520 nm. Representative TA spectra are collected at various delay times. **c** The B850 BChls fluorescence decay recoded after excitation at BChl *a* Q_x_ band at 590 nm. The profile can be successfully fitted with mono-exponential decay convoluted by instrument response (IRF) function

Figure 6 shows TA spectra of the spheroidenone-LH2 complex taken at different delay times in the visible-NIR (combined) range at RT (a) and at 77 K (b). The samples were excited in the carotenoid band at 520 nm. At early delay times (100 fs) the TA spectra show features characteristic of the S_2_ (1^1^B_u_^+^) state of spheroidenone, including GSB of the carotenoid absorption appearing simultaneously with GSB of the B800 and B850 bands of the BChl *a* LH2 components and the carotenoid S_2_ (1^1^B_u_^+^) → S_*n*_ ESA, visible as a positive band with maximum ~1050 nm and spanning spectral range from BChls Q_y_ band to ~1250 nm.

The simultaneous GSB of BChl *a* and carotenoid is indicative of ultrafast (<150 fs) energy transfer from the carotenoid S_2_ (1^1^B_u_^+^) state to both B800 and B850 forms of BChl *a*. The ESA band of the carotenoid S_1_ (2^1^A_g_^−^) state emerges after several hundred femtoseconds, with a pronounced maximum at 611 nm. The band is broad, gradually decreasing in intensity to longer wavelength and extending to 780 nm. At 77 K, the ESA becomes more resolved and consists of two bands at 612 and 763 nm. In addition, at 77 K the strength of the S_2_ (1^1^B_u_^+^) → S_*n*_ TA band (~1050 nm) is apparently reduced as indicated by the amplitude relative to the carotenoid-derived TA bands (Fig. 6b vs. a). Not clearly perceptible in this LH2 system are two spectral features characteristic of carotenoids bound to LH2 complexes, which are a transient radical cation absorption band (usually appearing at ~1000 nm within few first picoseconds), and a transient absorption band associated with the so-called S* state (usually appearing at the short wavelength edge of the main S_1_ (2^1^A_g_^−^) → S_*n*_ ESA band) (Cong et al. 2008; Papagiannakis et al. 2002; Polivka et al. 2004). The S* transient spectral feature has not been reported previously in this particular LH2; however, the spheroidenone radical cation has been reported by Cong et al. (Cong et al. 2008). It is not clear whether the transient band in the NIR spectral range, assigned to the carotenoid cation absorption, was made correctly. The global fitting results of the NIR TA data of this LH2 performed in Cong et al. (2008) show no spectro-kinetic component (EADS) that has a lifetime longer than the carotenoid S_1_ state in LH2 (as in LH2 carotenoid cation typically does) and has spectral signature characteristic for carotenoid cation absorption band.

### Dynamics of B850* fluorescence

The profile of the fluorescence decay of the B850 BChls taken at RT is given in Fig. 5c. The kinetic trace was obtained upon excitation at 590 nm, corresponding to the Q_x_ band of BChl *a*. The ~1.4 ns time constant for emission decay is typical for LH2 with short-conjugated carotenoids like neurosporene or spheroidene that are not able to quench the lowest singlet excited state of the B850 BChls, as demonstrated in previous studies (Dilbeck et al. 2016).

### Target analysis of the spheroidenone-LH2 TA data

**Fig. 7:**
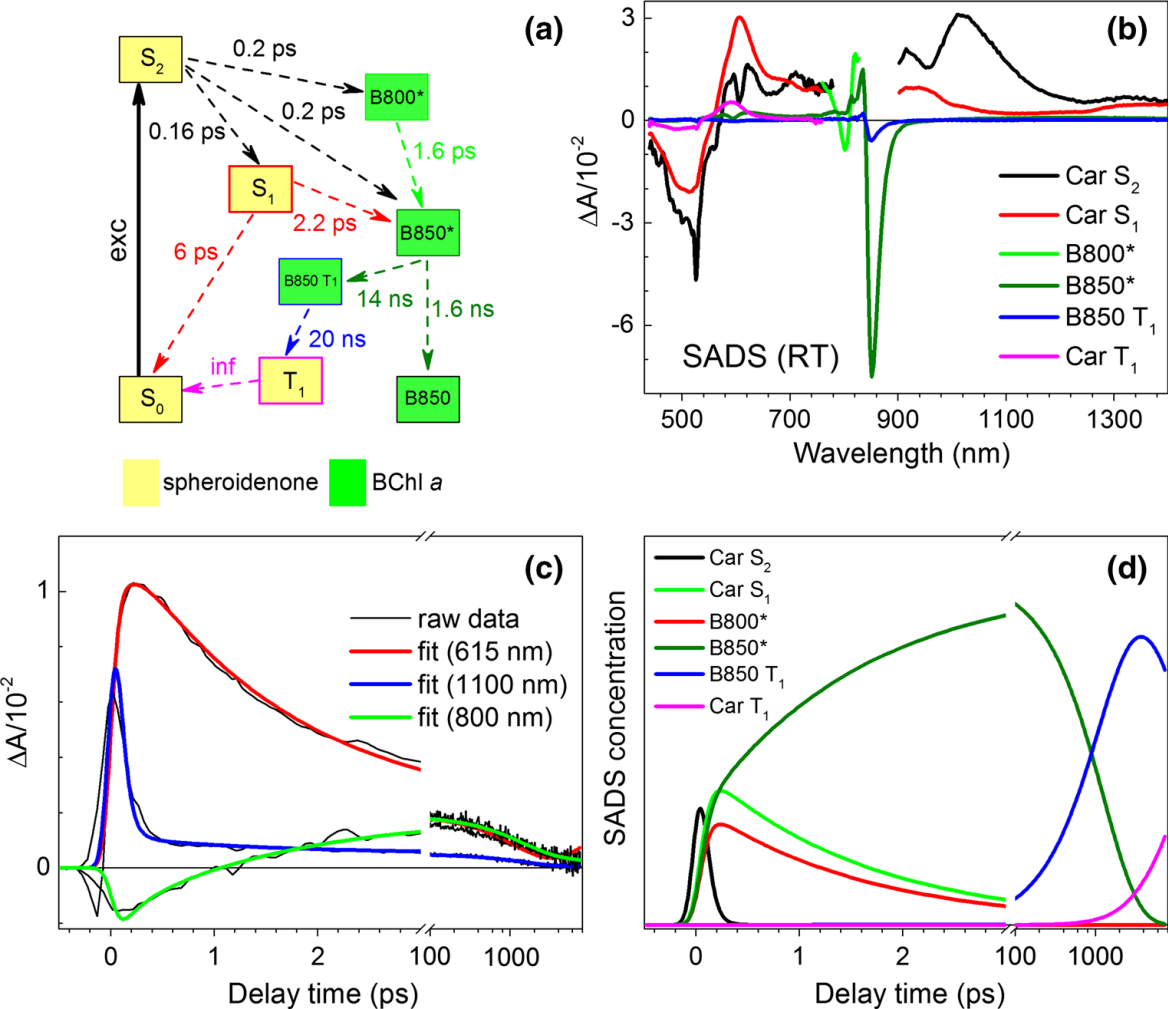
Target analysis of the LH2 TA dataset taken at RT. **a** The kinetic scheme of excitation decay path used for targeted kinetic analysis of the TA data, **b** SADS obtained from target analysis. In some cases, in order to avoid interaction between components with similar dynamics spectral constrains were applied. For more detail refer to the main text. **c** Exemplary kinetic traces and corresponding fits and **d** SADS concentrations demonstrating at which time delay specific SADS appears and the contribution to the observed TA spectrum

In order to gain further insight into the excited-state decay pathways after pumping the LH2-bound spheroidenone, kinetic modeling (target analysis) of the TA dataset collected at RT was performed. The decay scheme applied in the fitting procedure is shown in Fig. 7a. This kind of analysis requires prior knowledge of all decay rates and is useful to test specific kinetic models. The microscopic decay rate constants of the spheroidenone excited states (displayed as microscopic time constants) were deduced based on differences between intrinsic lifetimes of the excited states in the solvent(s) and effective lifetimes in the LH2. Importantly, the modeling should reproduce the overall *Φ*_Car→BChl_ of 92% obtained from the steady-state fluorescence excitation versus 1 − *T* measurement (Fig. 1c). In order to fulfil this requirement, a substantial part of excitation has to be routed from the S_2_ (1^1^B_u_^+^) state directly to BChls. This route is possible via resonance energy transfer from the spheroidenone S_2_ (1^1^B_u_^+^) excited state to the Q_x_ excited state of the BChl *a* molecules (that comprise B800 and B850). For simplicity, it was assumed that excitation energy transferred to BChl *a* via this process is split equally between B800 and B850 BChls. The intrinsic lifetime of the S_2_ (1^1^B_u_^+^) state has been reported to span 120–200 fs (Niedzwiedzki et al. 2011a; Zigmantas et al. 2004), from which a midpoint value of 160 fs was chosen. The time constant for B800 → B850 energy transfer utilized the measured *τ*_B800_ = 1.6 ps obtained from a single-exponential fit of the kinetic profile trace at 800 nm, assuming an efficiency of 100% for this process; a value on this order is consistent with prior results (Herek et al. 2000; Ma et al. 1997; Pullerits et al. 1997; Scholes and Fleming 2000; Wu et al. 1996).

The 1.4-ns lifetime of B850* was decomposed into intrinsic decay to the ground state (radiative and non-radiative decay) and to intersystem crossing (ISC) with time constants of 1.6 and 14 ns, respectively. These estimated values, which were based on a yield of ~10% (efficiency) for the BChl triplet calculated from TA spectra, are in reasonable agreement with *τ*_ISC_ ≈ 7 ns (8% efficiency) obtained previously for spheroidene-LH2 (Kosumi et al. 2016). The B850 triplets are ultimately quenched by spheroidenone triplets with an anticipated time constant of ~20 ns, in agreement with the recently obtained lifetime of ~17 ns for the spheroidene-LH2 protein (Kosumi et al. 2016). Finally, the carotenoid triplet was assumed to decay with infinite lifetime (for this time window). Only 30% of the total excitation is transferred to BChls through the S_1_ (2^1^A_g_^−^) state in order to achieve consistency with the overall *Φ*_Car→BChl_ of 92% from the steady-state fluorescence excitation versus absorptance measurements.

Spectral constraints were applied to account for the fact that some molecules should mainly contribute in certain spectral ranges (e.g., B800 BChl *a* mostly contributes around 800 nm). The introduction of such constraints was necessary to obtain sensible results; however, their incorporation into the whole spectral range could affect other components with similar lifetimes (e.g., B800* and spheroidenone S_1_ (2^1^A_g_^−^) state). The results of the target analysis, SADS, and their concentrations based on the kinetic scheme from Fig. 7a are given in Fig. 7b, c with legends assigning specific molecular species. The quality of the simulation with measured data is shown in Fig. 7d, which shows representative measured kinetic traces together with corresponding simulated traces obtained from the model.

### Spectral reconstruction of spheroidenone absorption in LH2

**Fig. 8:**
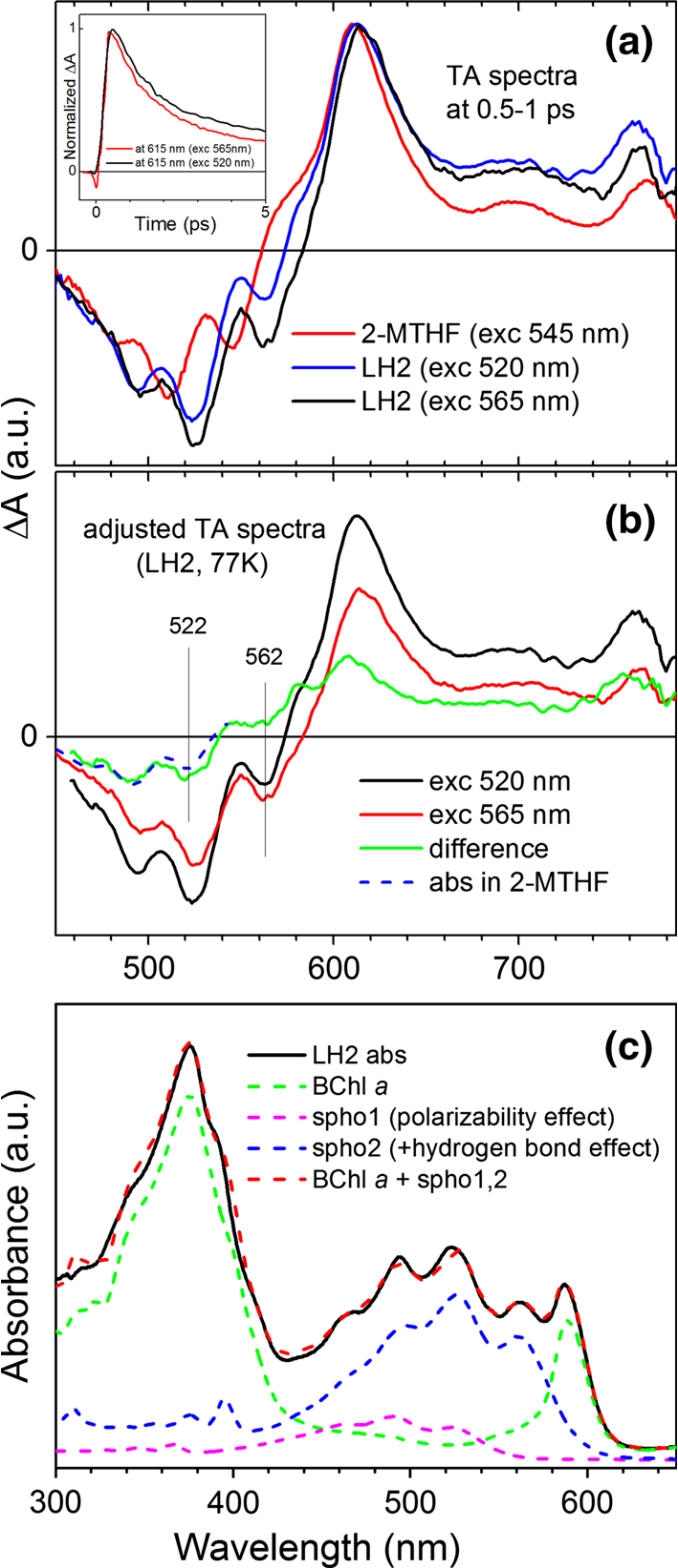
Two spectral forms of spheroidenone in the LH2 complex. **a** Exemplary TA spectra of spheroidenone in various environments and excitation wavelengths. The spectra are normalized at the maximum of the main ESA band. Note differences in the bleaching region and substantial similarities of the ESA bands. The *insert* shows dynamics of spheroidenone S_1_ → S_*n*_ transient band upon both excitations. The temporal characteristics of decays are not very different. **b** Reconstruction of contribution of the “*blue*” form of spheroidenone in the 520 nm TA spectrum (*green*). The bleaching of “*blue*” spheroidenone absorption overlaid with spheroidenone absorption taken at 77 K in 2-MTHF (*dashed, blue*) (after adequate shift and reflection) reveals good match in a spectral shape. The (0–0) vibronic band in both spectral form of spheroidenone is indicated. **c** Spectral reconstruction of the carotenoid band in 77 K steady-state absorption of the spheroidenone-LH2. Two spectral forms of spheroidenone mimicked by the absorption spectrum taken at 77 K in 2-MTHF and adequately shifted in energies

Scrutiny of the TA data taken at 77 K in organic solvent and in the LH2 suggests that upon binding into the protein the carotenoid differentiates into two spectral forms, shown in Fig. 8. Figure 8b shows three exemplary TA spectra of spheroidenone recorded under various conditions. Two spectra are taken for the LH2 using two different excitation wavelengths, 520 and 565 nm. The spectra are normalized at the maximum of the ESA band. Interestingly, the spectra show that the ESA bands essentially overlap, although the GSBs do not have the same amplitude. This result is most simply explained if the excitation wavelengths are selective and each predominantly excites a different spectral form of the molecule. It may be surprising that different spectral forms of the same carotenoid will reveal essentially the same excited state properties such as nearly identical ESA bands. However, it is possible if only the S_2_ (1^1^B_u_^+^) state is perturbed but other states are not. The TA spectrum of spheroidenone taken in 2-MTHF (Fig. 8a, red) at 77 K overlaid with those taken in LH2 at different excitation wavelengths shows this is likely the case, as the ESA bands also overlap though the GSB is substantially shifted to shorter wavelength relative to the same carotenoid in LH2 (for two different excitation wavelengths). If 520 and 565 nm excitations produce two spectral forms of spheroidenone with different weights, it is possible to separate them from each other. Such spectral reconstruction shown in Fig. 8b reveals “blue” spheroidenone at 522 nm. The major spectral form is shifted to longer wavelengths by 40 nm, to give the main S_2_ (1^1^B_u_^+^) (0,0) vibronic band of “red” spheroidenone at 562 nm (vide supra). Lastly, this information can be used to reconstruct the carotenoid absorption contour observed in 77 K steady-state absorption of spheroidenone-LH2, using an adequately shifted (due to medium polarizability differences) spheroidenone absorption spectrum recorded in 2-MTHF. The reconstruction is shown in Fig. 8c.

## Discussion

### Excited-state properties of spheroidenone in solvents and in LH2

Spheroidenone has been studied in a few polar and nonpolar solvents (Cong et al. 2008; Frank et al. 2000; Niedzwiedzki et al. 2011a; Zigmantas et al. 2004) as well as in light-harvesting proteins such as LH1 (Šlouf et al. 2012, 2013) and LH2 (Cong et al. 2008) from *Rba. sphaeroides* and in the so-called LH4 complex from *Roseobacter denitrificans*—the LH2 complex in which the B800 and B850 bands are degenerate and overlap at 800 nm (Niedzwiedzki et al. 2011a). In organic solvents, S_1_ (2^1^A_g_^−^) state dynamics are not sensitive to either a change in polarity or polarizability and the state lifetime has a constant value of ~6 ps (Cong et al. 2008; Frank et al. 2000; Niedzwiedzki et al. 2011a; Zigmantas et al. 2004). The present study shows that the lifetime is only slightly longer at cryogenic temperature (~9 ps). However, apparent differences in excited state properties appear upon binding the carotenoid into various LH systems. For spheroidenone in LH2, an effective lifetime of the S_1_ (2^1^A_g_^−^) state was reported to span a range of 800–900 fs, while in the LH1 the S_1_ (2^1^A_g_^−^) state decays with two kinetic components with lifetimes of 0.4 and 2.4 ps (Šlouf et al. 2012). The two components are longer in the LH4 complex, for which the carotenoid S_1_ (2^1^A_g_^−^) state decays with time constants of 0.8 and 5.2 ps (Niedzwiedzki et al. 2011a). The latter result suggests that the LH4 may contain a pool of carotenoid molecules that is not optimally bound in the binding pocket and is practically excluded from energy transfer to BChl *a*. Previous studies showed that the spheroidenone ESA band has a modified shape for LH1 versus LH2 complexes, suggesting structural alteration of double-bond conjugation (Šlouf et al. 2012). In organic solvents, the most stable form of spheroidenone is a structure with the carbonyl group in s-*cis* configuration as shown in Fig. 1 (Šlouf et al. 2012, 2013). However, it has been proposed that in the LH1 the carotenoid is altered to an s-*trans* structure that is probably stabilized by hydrogen bonding with a protein residue (Šlouf et al. 2012, 2013).

The combination of such factors along with the polarity of the carotenoid binding pocket in LH2 has been suggested to be responsible for alteration of the shape of ESA band such that the excited state responsible for spheroidenone in LH1 has ICT-like character, rather than simply being S_1_ (2^1^A_g_^−^) (Šlouf et al. 2012, 2013). The shape of the carotenoid ESA band for spheroidenone-LH2 complex is similar to that obtained in methanol. Furthermore, the shape of the ESA band for spheroidenone-LH2 at 77 K is comparable to that observed at 77 K in 2-MTHF. These results indicate that in the LH2 pigment–protein interactions do not modify the carotenoid structure with respect to that adopted in organic solvents and thus an ICT state cannot be formed due to negligible influence of the C=O bond on the rest of the double-bond conjugation. Assuming that the polarizability of the pigment environment is the primary factor driving its spectral properties, the spheroidenone (0–0) vibronic peak in the ground-state absorption spectrum is anticipated to be at ~520 nm (Fig. 3), yet is found at 562 nm (Fig. 1). Accordingly, new lines of thought are needed to reconcile these observations.

### Two spectral forms of spheroidenone in the LH2

Several issues raised above can be reconciled under the view that spheroidenone in the LH2 can adopt two forms, which were termed “blue” and “red” in the last Results section based on analysis of the TA and ground-state absorption data. The additional considerable bathochromic shift of “red” spheroidenone could be brought about by an electronic polarization effect, introduced by potential hydrogen bonding of the spheroidenone keto group with a protein amino acid residue. A bathochromic spectral shift caused by establishing a hydrogen bond with a solvent molecule was observed for another keto-bearing carotenoid, peridinin. In protic solvents, like methanol or ethylene glycol, two spectral species, so-called “normal” and “red” forms of the carotenoid, are formed and coexist (Zigmantas et al. 2003). That situation resembles what is seen for spheroidenone in the LH2. As noted above, hydrogen bonding of spheroidenone in the binding site of the LH1 complex of *Rba. sphaeroides* had been proposed (Šlouf et al. 2012). However, the best candidate for providing hydrogen bonding, a highly conserved Lys-Ile-Trp motif present in the N-terminal domain of the LH1 α-polypeptide, has been disqualified based on the argument that it is also present in the LH2 complex in which spheroidenone shows no sign of ICT character in its first excited singlet state (Šlouf et al. 2012). However, hydrogen bonding most likely will not lead to formation of an ICT state if simultaneously the –C=O group is not rotated to the s-*trans* configuration. This lack of simultaneous H-bonding/rotation is what most likely occurs in the spheroidenone-LH2, so although the major pool of carotenoids is involved in hydrogen bonding the pigment geometry is maintained. On the other hand, H-bonding makes the carotenoid molecule effectively more polarized and as a consequence, much more sensitive to the high polarizability of the binding pocket.

## Conclusions

In this study, we have demonstrated that the carotenoid absorption band in the spheroidenone-LH2 complex from *Rba. sphaeroides* is comprised of two spectral forms of the carotenoid. The minor “blue” spheroidenone with an origin at 522 nm is a spectral form induced by high polarizability of the binding site. The major “red” form at 562 nm is associated with a pool of pigments that interact with protein residues most likely via hydrogen bonding. This interaction increases the effective molecular polarizability and in turn provides an additional bathochromic absorption shift for the carotenoid in the LH2 protein pocket, which presents a strong permanent electric field to the carotenoid from nearby point charges, such as the positively charged βArg29 residue indicated from prior studies (Crielaard et al. 1994). Spectroscopic results show that “red” spheroidenones do not have an altered geometry of the double-bond conjugation and an ICT-like excited state cannot be formed following excitation. Application of target kinetic modeling of excited state decay pathways open to the carotenoid band suggests that very efficient *Φ*_Car→BChl_ of this LH2 system most likely derives from resonance energy transfer from spheroidenone S_2_ (1^1^B_u_^+^) state to BChl *a* via the Q_x_ band of the latter, accounting for 60% of the 92% total transfer. The elevated S_2_ (1^1^B_u_^+^) → Q_x_ transfer efficiency (over that expected for a carotenoid having the same overall conjugation length but provided only by C=C bonds) is apparently associated with substantially decreased energy gap (increased spectral overlap) between the hypothetical S_2_ (1^1^B_u_^+^) → S_0_ (1^1^A_g_^−^) spheroidenone emission and Q_x_ absorption of BChl *a*. This reduced energy gap is the ultimate consequence of the inferred carotenoid–protein hydrogen bonding. This structural motif is likely a key factor underlying the surprisingly high overall *Φ*_Car→BChl_ in this LH2 antenna complex.

## Abbreviations

2-MTHF: 2-Methyltetrahydrofuran
BChl: Bacteriochlorophyll
EADS: Evolution-associated difference spectra
ESA: Excited state absorption
Exc: Fluorescence excitation
FWHM: Full width at half maximum
GSB: Grounds state absorption bleaching
ICT: Intramolecular charge transfer
IRF: Instrument response function
ISC: Intersystem crossing
LDAO: Lauryl dimethylamine-oxide
LH1: Light-harvesting complex 1
LH2: Light-harvesting complex 2
NIR: Near infrared
OD: Optical density
RC: Reaction center
RR: Resonance Raman
RT: Room temperature
SADS: Species-associated difference spectra
TA: Transient absorption
TCSPC: Time-correlated single-photon counting
THF: Tetrahydrofuran
